# SoleFusion-Net: an explainable multimodal deep learning framework for diabetic foot syndrome classification in type II diabetes mellitus

**DOI:** 10.1038/s41598-026-42207-6

**Published:** 2026-04-03

**Authors:** Mehewish Musheer Sheikh, Mamatha Balachandra, Narendra V. G, Arun G. Maiya

**Affiliations:** 1 Manipal Institute of Technology, Manipal Academy of Higher Education, Manipal, 576104 India; 2 Department of Physiotherapy, Manipal College of Health Professions, Manipal Academy of Higher Education, Manipal, 576104 India

**Keywords:** Diabetic foot syndrome, multimodal deep learning, plantar pressure imaging, neuropathy classification, Explainable Artificial Intelligence, Computational biology and bioinformatics, Engineering, Health care, Mathematics and computing, Medical research

## Abstract

Early detection of diabetic neuropathy remains a challenge due to reliance on subjective clinical assessments. We propose SoleFusion-Net, an explainable multimodal deep learning framework that integrates plantar pressure images with structured clinical data through an late-fusion dual-branch architecture. The image branch employs convolutional layers to extract biomechanical pressure features, while the tabular branch processes clinical and demographic variables. Learned representations are fused for joint classification. Extensive explainability techniques, including Grad-CAM, SHAP, surrogate decision trees, and prototyped critique analysis, were implemented to enhance transparency and clinician trust. Evaluated on 504 patients stratified into mild, moderate, and severe neuropathy, SoleFusion-Net achieved 83% validation accuracy with AUCs of 0.962, 0.892, and 0.933 across the three classes. Explainability analyses identified vibration perception threshold and monofilament scores as critical predictors, while Grad-Cam highlighted spatially significant plantar regions. SoleFusion-Net showcases the feasibility of adjusting the standard multimodal fusion process and the use of various XAI methods to achieve the goal of diabetic foot syndrome classification. Therefore, this solution ensures a trustworthy help about risk stratification. The framework provides a modular and explainable AI model for integrating multimodal biomedical data, with a structure that is potentially generalizable to other settings pending external validation.

**Supplementary Information:**

The online version contains supplementary material available at 10.1038/s41598-026-42207-6.

## Introduction

Diabetes is a chronic metabolic disorder characterized by persistent hyperglycemia resulting from inadequate insulin production or utilization, leading to long-term complications such as cardiovascular disease, nephropathy, retinopathy, and neuropathy. Among these, diabetic peripheral neuropathy (DPN) is a major cause of foot ulceration and amputation, significantly reducing quality of life.

Globally, over 589 million people are affected by diabetes, with Type 2 Diabetes Mellitus (T2DM) accounting for more than 90% of cases^1^. South Asia faces a particularly high disease burden, and India alone hosts one in seven adults with diabetes^2^. Indian studies report a wide DPN prevalence range (9.6–78%), reflecting heterogeneous diagnostic criteria and limited early screening infrastructure.

Conventional diabetic foot assessment relies on subjective tests—such as monofilament and vibration perception threshold (VPT)—that suffer from inter-observer variability and poor sensitivity to subclinical neuropathy^3,4^. Recent work highlights plantar pressure distribution as an objective, quantitative biomarker for neuropathic risk^5^. Plantar pressure imaging captures subtle gait and biomechanical deviations that often precede ulceration^6,7^.

Machine learning (ML) and deep learning (DL) have transformed such biomechanical analyses by learning diagnostic representations directly from data^8–13^. However, existing ML pipelines depend heavily on high quality, homogeneous datasets and often generalize poorly across populations^9,10^. Ethical and privacy concerns further limit multi-centre data aggregation. DL models alleviate these constraints through automated feature extraction and improved robustness^11–13^, yet interpretability remains a major barrier to clinical trust and adoption.

Multimodal data integration—combining imaging and structured clinical inputs—has emerged as a promising approach in precision medicine^14–16^. Nevertheless, most diabetic neuropathy studies still employ unimodal or late-fusion models^17–20^. Earlier CNN-based works using plantar pressure or thermal imaging achieved accuracies exceeding 95%^17,21–26^ but lacked clinical context. Hybrid frameworks combining clinical variables and sensor data (e.g., temperature, pressure, gait)^22,23,27,28^ demonstrated feasibility but offered limited interpretability and generalization.

The latest development in XAI has given notice to and made clear the necessity of using transparent and interpretable models in medical practice. Thorough assessments, like the one done by Aqib Nazir et al.^29^, display the medical image analysis society’s increasing reliance on XAI and reveal model interpretability as a key factor for clinical acceptance. In the same way, the state-of-the-art multimodal learning, such as transformer-based architectures and self-alignment frameworks, is already promising new directions for the integration of diverse data sources^30,31^. Recent examples such as CURENet (Doa et al.^32^ have shown the success of overarching multimodal representations for chronic disease prediction, thus supporting the use of combined clinical and physiological modalities.

Despite progress in planar pressure analysis and machine learning, existing approaches often remain limited to single modalities or rely on late-stage fusion, which restricts their diagnostic power and interpretability. This raises a critical research question: C*an a late-fusion multimodal deep learning framework that jointly learns plant repressure images and clinical data improve both accuracy and explainability in peripheral neuropathy diagnosis?* Our objective is to develop such a framework to address these limitations.

In response to these constraints, we introduce SoleFusion Net, a new multimodal deep learning framework that uses a late fusion architecture to integrate plantar pressure images with clinical data. Joint feature learning from both modalities is made possible by our dual-branch architecture, which captures intricate relationships between clinical traits and biomechanical patterns. The system includes extensive explainability tools, such as SHAP for clinical feature importance analysis and Grad-Cam for visual pressure pattern interpretation.

The following significant contributions have been made:

1. Introducing a novel late fusion architecture enabling joint representation learning from plantar pressure images and clinical data.
2. Demonstrating superior classification performance over a single-modality baseline by evaluating 504 patients with varying neuropathy severity.
3. Providing extensive interpretability analysis, making decision-making transparent and clinically meaningful.

Though dual-branch multimodal architectures and tools like Grad-CAM and SHAP from the XAI category have been employed in different medical areas, the combined use of these techniques for diabetic foot syndrome is still a relatively unexplored area, particularly in the case of combining plantar pressure images with structured clinical data. Hence, our research modifies and authenticates this well-known technical framework to solve a major clinical problem, while also highlighting clinical explainability and usability.

The clinical significance extends beyond improved accuracy. SoleFusion-Net provides objective, interpretable assessment tools supporting informed clinical decisions, facilitating early intervention strategies, and potentially reducing morbidity and healthcare costs associated with diabetic foot complications. The framework’s modular design makes it structurally adaptable to different clinical settings and extensible to other multimodal diagnostic tasks, though its clinical generalizability requires validation in multi-centre studies.

## Method

### Study design

**Fig. 1:**
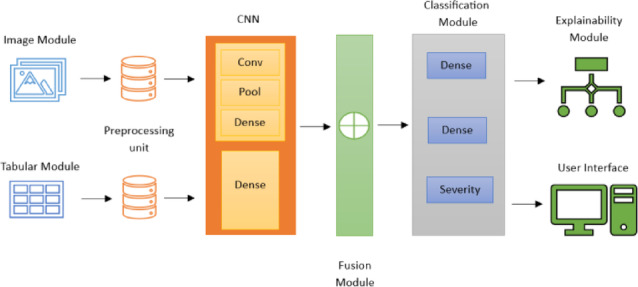
Architecture of the proposed model.

This section describes our techniques for classifying diabetic foot syndrome utilising plantar pressure images and clinical and demographic tabular data to take advantage of complementary information from both modalities. This study uses a retrospective analysis design to create and assess a multimodal deep learning framework for classifying foot severity. The main reason for merging these data sources is that. In contrast, clinical factors such as age, BMI, etc., offer crucial contextual information that might not be immediately obvious; visual patterns in plantar pressure distribution may be able to identify biomechanical anomalies. Using a dual-branch neural network to handle image and tabular inputs simultaneously and combine their learnt representations, the model seeks to improve predictive performance over unimodal methods as shown in Fig. 1.

### Dataset description

The Win-Track system (MEDICAPTURES Technology, France) was used to record plantar pressure and gait characteristics, with automatic footsteps recognition and parameter computation performed after data transfer to a computer. Quantitative gait data were provided to the clinician through this system. The dataset comprised plantar pressure images and corresponding demographic and clinical records collected at the Physiotherapy Department, Kasturba Medical College, Manipal. Annotation was performed using vibration perception threshold (VPT) as the primary criterion, stratifying patients into three neuropathy severity categories, as shown in Table 1.

**Table 1:** Dataset description.

Neuropathy severity	VPT range (volts)	No. of patients
Mild	15–24	186
Moderate	25–39	172
Severe	> 40	146
Total	–	504

Modality of images, to maintain relative intensity patterns, each plantar pressure image was pre-processed to a consistent 224 × 224-pixel size. Images were taken under control to guarantee uniform foot positioning and load distribution. Regarding Tabular modality, age, sex, body mass index (BMI), length of symptoms, and pertinent comorbidities were among the clinical and demographic characteristics included in the structured dataset. Categorical variables were label-encoded, while continuous variables were standardized using z-score normalization. The dataset showed a somewhat unbalanced class distribution with 36.9% mild, 34.1% intermediate, and 29% severe cases. A unique patient identification was used to link each image to its accompanying clinical record, guaranteeing precise multimodal pairing for model training.

### Data preprocessing and augmentation

To enhance model generalization and mitigate overfitting, separate preprocessing pipelines were designed for the image and tabular modalities.

1. Image Preprocessing.

Each plantar pressure image was scaled to 224 × 224 × 3 and adjusted to the range [0,1]. During training, augmentation (used to improve generalization and combat overfitting) was employed online to simulate the real-world variability in patient foot placement and measurement noise. The pipeline segmentation comprised geometric operations: random cropping, flipping in horizontal and vertical directions, and small-angle rotations (± 15). Gaussian noise injection and arbitrary brightness and contrast shifts are examples of photometric adjustments. Colour disturbances include grayscale conversion (random probability), histogram equalisation, and HSV channel shifting, as shown in Supplementary Fig. S1. Because these processes were implemented as bespoke TensorFlow layers, they were compatible with GPU acceleration and reproducible.

(b) Tabular data preprocessing.

The image modality was not used to process the tabular clinical features. Categorical variables were label-encoded, while continuous variables were standardized using z-score normalization. For continuous variables, the median was used to impute missing values; for categorical variables, a specific placeholder category was used. Only records with valid plantar pressure images and complete clinical data were retained to ensure modality correspondence. All preprocessing steps were applied after stratified data splitting to prevent information leakage.

### Model architecture

**Fig. 2:**
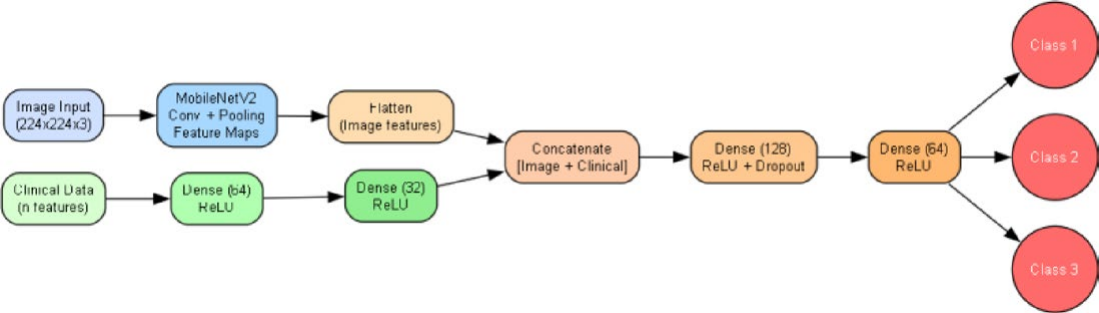
Architecture overview of the proposed network.

A custom multimodal deep learning architecture was developed to process plantar pressure images and corresponding clinical tabular features jointly. The model followed a dual-branch design, with independent feature extraction pipelines for each modality and a late-fusion strategy for decision-making, as shown in Fig. 2.

1. Image branch (convolutional neural network).

Patterns of spatial pressure distribution were intended to be captured by the image route. The branch started with a data augmentation block that included Gaussian noise injection, rotation, zoom, brightness/contrast variation, flipping, rescaling, and random cropping.

**Fig. 3:**
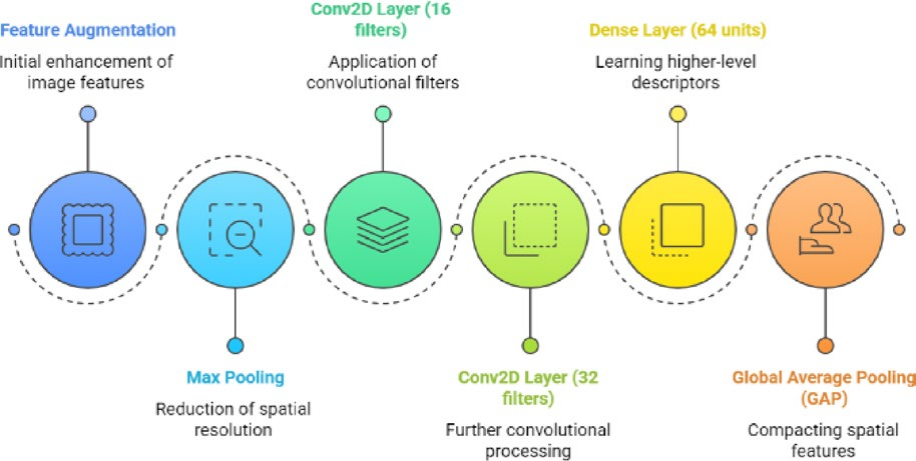
Pipeline of the image branch.

After augmentation, the features were recovered using max pooling and the Conv2D layer (16 filters, 3 × 3, ReLU activation) with L2 regularization. The last convolutional block, the Conv2D layer (32 filters, 3 × 3, ReLU activation), with L2 regularization, is followed by a max pooling layer to reduce spatial resolution while retaining essential features progressively. A Dense layer (64 units, ReLU) is employed to learn higher-level image descriptors using dropout for regularization and Global Average Pooling (GAP) to reduce spatial features into a compact representation. The pipeline for the Image branch is shown in Fig. 3. This branch effectively learns localized pressure distribution patterns clinically indicative of foot biomechanics and pathology.

(b) Tabular branch (fully connected network).

**Fig. 4:**
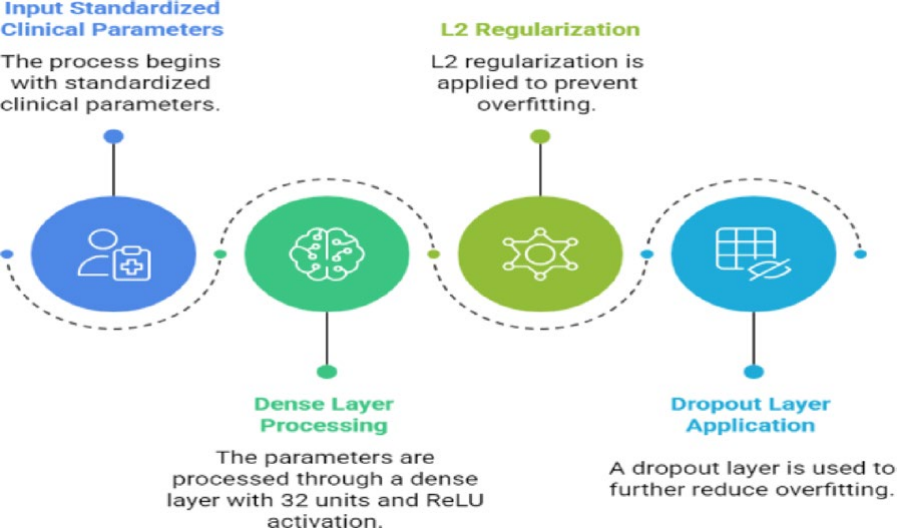
Pipeline of the tabular branch.

The tabular pathway processed standardized clinical parameters. It consisted of two layers: a Dense layer (32 units, ReLU) with L2 regularization and a Dropout layer to reduce overfitting as shown in Fig. 4. This branch extracts latent representations of structured clinical information that may not be visible in plantar pressure images alone.

(c) Multimodal fusion and classification.

**Fig. 5:**
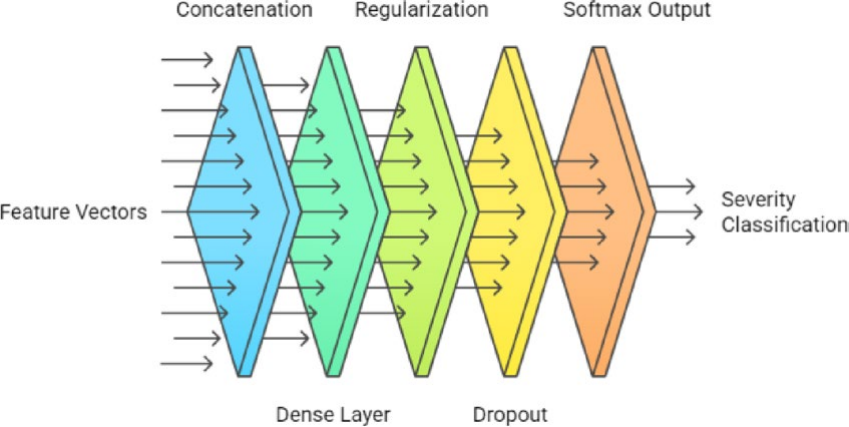
Pipeline for classification.

The feature vectors from both branches were concatenated and passed through a Dense layer (32 units, ReLU) with L2 regularization and dropout. SoftMax output layer that produces probability distributions across the target classes has three neurons corresponding to the severity classes: Mild, Moderate, and Severe. The pipeline for the classification module is shown in Fig. 5. This fusion enables the model to learn complementary visual and clinical feature relationships.

The late fusion process can be mathematically represented as:

$$\widehat{y}=g\left({f}_{img}\right({X}_{img})\oplus{f}_{tab}({X}_{tab}\left)\right)$$

where ${X}_{img}$ is the plantar pressure image, ${X}_{tab}$​ is the clinical/tabular data, ${f}_{img}$​(·) and ${f}_{tab}$(·) represent the learned feature extraction function for each modality, ⊕ denotes concatenation, and g(·) is the fusion and classification network.

(d) Optimization.

The categorical cross-entropy loss function, which assesses the difference between the actual labels and the predicted probability distribution, was used to train the model. This function is suitable for multiclass classification tasks. The Adam optimizer was used for optimization because of its adaptive learning rate mechanism, which speeds up convergence while preserving stability with varying parameter updates. Several callbacks were used to guarantee effective and reliable training, such as early stopping, which prevented overfitting by terminating training when the validation loss stopped improving after seven epochs. Model checkpointing was used to save the best-performing model in each cross-validation fold, guaranteeing that only the most generalizable weights were kept. ReduceLROnPlateau was used to automatically lower the learning rate when the validation loss plateaued, allowing the model to fine-tune its convergence in later stages of training. In fact, early stopping frequently ended training earlier once ideal validation performance was attained, striking a balance between accuracy and efficiency. Training was carried out for a maximum of 50 epochs with a batch size of 8–16.

For reproducibility, the architectural and training details are provided here. The image branch consisted of two convolutional blocks: the first Conv2D layer, followed by MaxPooling2D; then a second Conv2D layer and another MaxPooling2D layer. Subsequently, global average pooling was applied, followed by a dense layer with 64 units, ReLU activation, and dropout (rate = 0.5). The tabular branch consisted of a single dense layer with 32 units, ReLU activation, L2 regularization, and dropout (rate = 0.3). The following feature concatenates a fusion dense layer integrated multimodal representations before the final three-unit SoftMax classification layer.

(e) Training strategy.

The proposed multimodal model was trained and evaluated using a 5-fold stratified cross-validation scheme to ensure robust performance estimation and reduce the risk of overfitting. This approach preserved the class distribution across folds and allowed performance assessment across multiple train–validation splits.

The dataset was split into 80% training and 20% validation for each cross-validation fold, using stratified sampling to guarantee equal representation of each class. Only samples with complete clinical feature sets and genuine plantar pressure images were used in the training to ensure data quality and consistency. The Adam optimizer was used to train the model with a batch size 16, a maximum of 50 epochs, and categorical cross-entropy loss. The best-performing model weights were restored for evaluation when early stopping was implemented to end training when no more validation improvement was seen automatically.

The model is optimized using categorical cross-entropy loss:

$$\mathrm{L}=-\sum_{c=1}^{3}{y}_{c}\cdot\mathrm{l}\mathrm{o}\mathrm{g}\left({\widehat{y}}_{c}\right)$$

where ${y}_{c}$ is the ground truth label for class c and ${\widehat{y}}_{c}$ is the predicted probability for that class.

A variety of regularization techniques were used to reduce overfitting and enhance generalization. The image branch improved the model’s capacity to manage data variability input by using random geometric and photometric changes for data augmentation. Dropout layers were added to the image and tabular branches during the fusion stage to lessen neuronal co-adaptation. L2 weight regularization was also applied to important convolutional and dense layers to prevent over-complexity in the learned parameters.

A learning rate scheduling technique that used the ReduceLROnPlateau callback to halve the learning rate if the validation loss did not improve over three consecutive epochs greatly helped training stability. Early halting was initiated when validation loss remained constant for seven consecutive epochs to ensure adequate training without requiring too much computing. A thorough assessment of the model’s resilience was provided by reporting the mean and standard deviation of the training and validation accuracy and loss metrics for each fold and the overall model performance across all folds. The flowchart for this process is shown in Supplementary Fig. S2.

### Evaluation and explainability

The trained multimodal model was comprehensively evaluated using quantitative performance metrics and qualitative interpretability analyses.

1. Quantitative evaluation.

Accuracy, the correct classification rate, was calculated by comparing the model’s predictions for each fold with the validation set’s ground truth labels. Each class’s precision, recall, and F1-score enable evaluation of performance unique to that class. Normalized to percentages, the confusion matrix shows class-wise prediction trends and highlights common misclassifications. The confusion matrix was supplemented by analyzing the most confused class pairs and high-confidence misclassifications (predictions with ≥ 80% confidence but incorrect), revealing systematic model errors.

(b) Confidence analysis.

The average SoftMax confidence for correct and incorrect predictions was computed separately to evaluate model calibration. This helped determine whether high confidence reliably indicated correct classification or was associated with overconfident errors.

(c) Explainable AI (XAI) tools.

A suite of explainable artificial intelligence (XAI) techniques was applied to enhance the interpretability of the proposed multimodal deep learning framework, targeting both the image and tabular modalities and the combined decision process.

1. SHAP (Shapley Additive exPlanations).

By calculating Shapley values, SHAP allocates an importance value to each feature. Applying SHAP to the tabular branch allowed us to rank features globally and provide local explanations for individual forecasts in our work. This made it possible to determine which clinical factors impacted classification results most.

(2) LIME (Local Interpretable Model-Agnostic Explanations).

LIME uses an interpretable surrogate to imitate the complicated model locally. LIME identifies the attributes most important for a particular choice by varying inputs and tracking prediction changes. This made patient-specific forecasts interpretable at the instance level.

(3) Rule-based Surrogates.

Training surrogate models based on decision trees approximated the deep learning system’s behaviour. Clinicians can better comprehend model logic thanks to the clear, understandable decision paths created by the rules. These explanations based on regulations serve as a tool for global interpretability.

(4) Prototype and criticism-based explanations.

Using embedding space distances, representative samples (prototypes) and atypical examples (criticisms) were distinguished. While critics point out anomalies or edge cases, prototypes show the model’s " typical " instances. His approach supports clinical decision-making procedures by using case-based reasoning.

(5) Grad-CAM (Gradient-weighted Class Activation Mapping).

Grad-CAM creates heatmaps over input images when applied to the convolutional branch of the model, showing which regions had the most significant impact on the prediction. This visual interpretability, which indicates where the network “looks” while classifying plantar pressure images, is essential for the image modality.

Grad-Cam heatmaps are computed as:

$${L}_{Grad-CAM}^{k}=\mathrm{R}\mathrm{e}\mathrm{L}\mathrm{U}\left(\sum\limits_{l}{\alpha}_{k}^{l}{A}^{l}\right),{\alpha}_{k}^{l}=\frac{1}{Z}\sum\limits_{i,j}\frac{{\partial y}^{k}}{{\partial A}_{i,j}^{l}}$$

where ${A}^{l}$ are the features maps from layer l, and ${y}^{k}$ is the score for class k.

## Results

### Model performance

Using 5-fold stratified cross-validation, the suggested multimodal model was assessed to guarantee a reliable and objective performance estimate. The model had an average validation loss of 0.7515 ± 0.02 and an average validation accuracy of 83.0% ± 2.1% across folds. The model demonstrates internal consistency and low variance across cross-validation folds, indicating robustness within the study’s cohort. However, external validation is necessary to assess true generalizability across diverse populations and clinical settings.

**Fig. 6:**
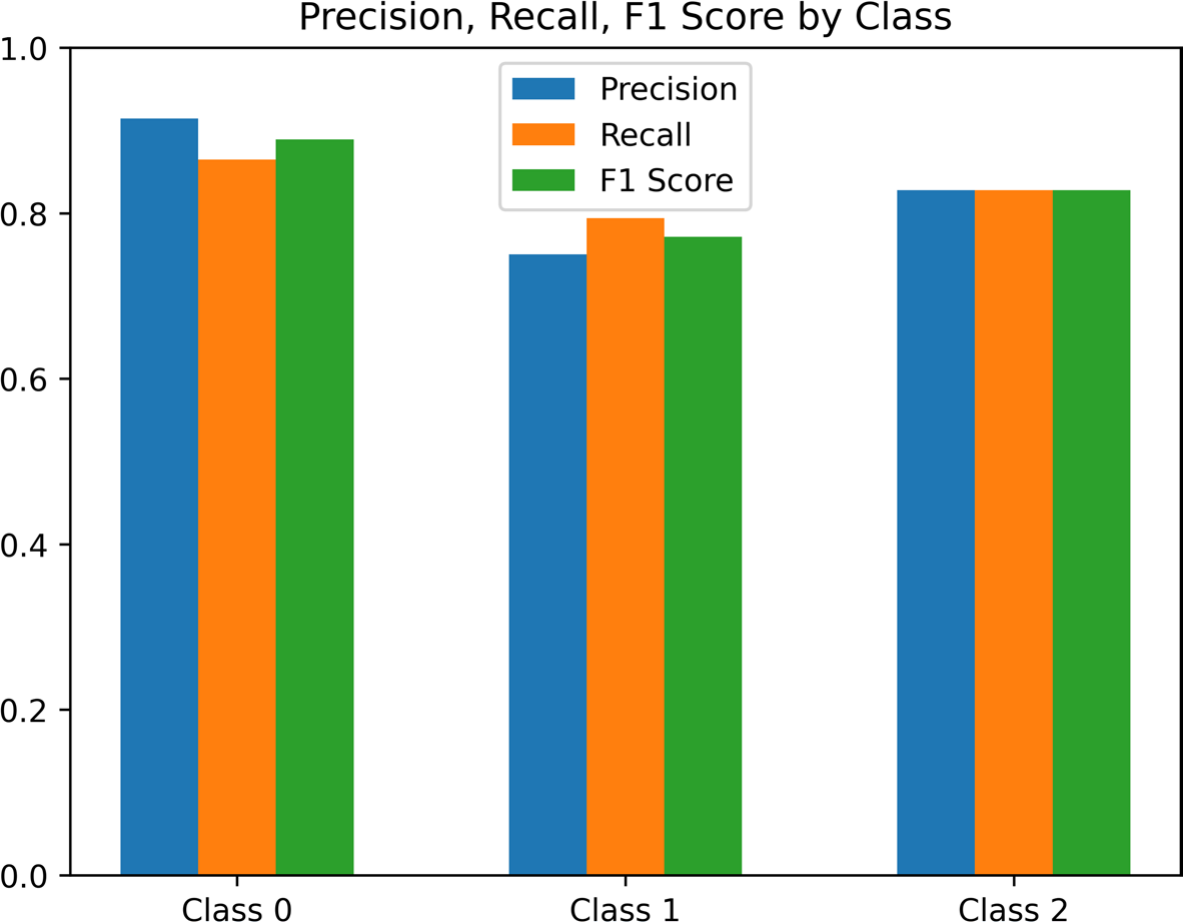
Class-wise metric evaluation of the model in a bar graph.

During training, the model achieved a final training accuracy of 87.4% with a corresponding training loss of 0.6992, while the final validation accuracy was 83.0% with a validation loss of 0.7515. The observed training validation accuracy gap of ~ 4.4% indicates mild overfitting, which was mitigated using regularization (dropout, L2 weight decay), data augmentation, and early stopping. The validation performance stabilizing near 82–85% highlights the model’s ability to balance learning capacity with generalization. Metric evaluation is shown in Fig. 6, and a class-wise classification report is also given in Table 2. The results below show that the multimodal technique successfully combines tabular clinical information with plantar pressure images for categorization.

**Table 2:** Class-wise classification report.

Class/metric	Accuracy (%)	Precision (%)	Recall (%)	F1 score (%)
Class 0	86.5	91.4	86.5	88.9
Class 1	79.4	75.0	79.4	77.1
Class 2	82.8	82.8	82.8	82.8

Cross-validation and regularization ensure the model maintains good generalization and stays clear of extreme overfitting, even while training accuracy is higher than validation accuracy. The overfitting analysis revealed a minimal train-validation gap ~ 4.4%, confirming that the model demonstrates strong generalization capacity without significant overfitting. Model accuracy across epochs, Model Loss across Epochs, and Learning rate adjustment during training, as shown in Supplementary Fig. S3.

**Fig. 7:**
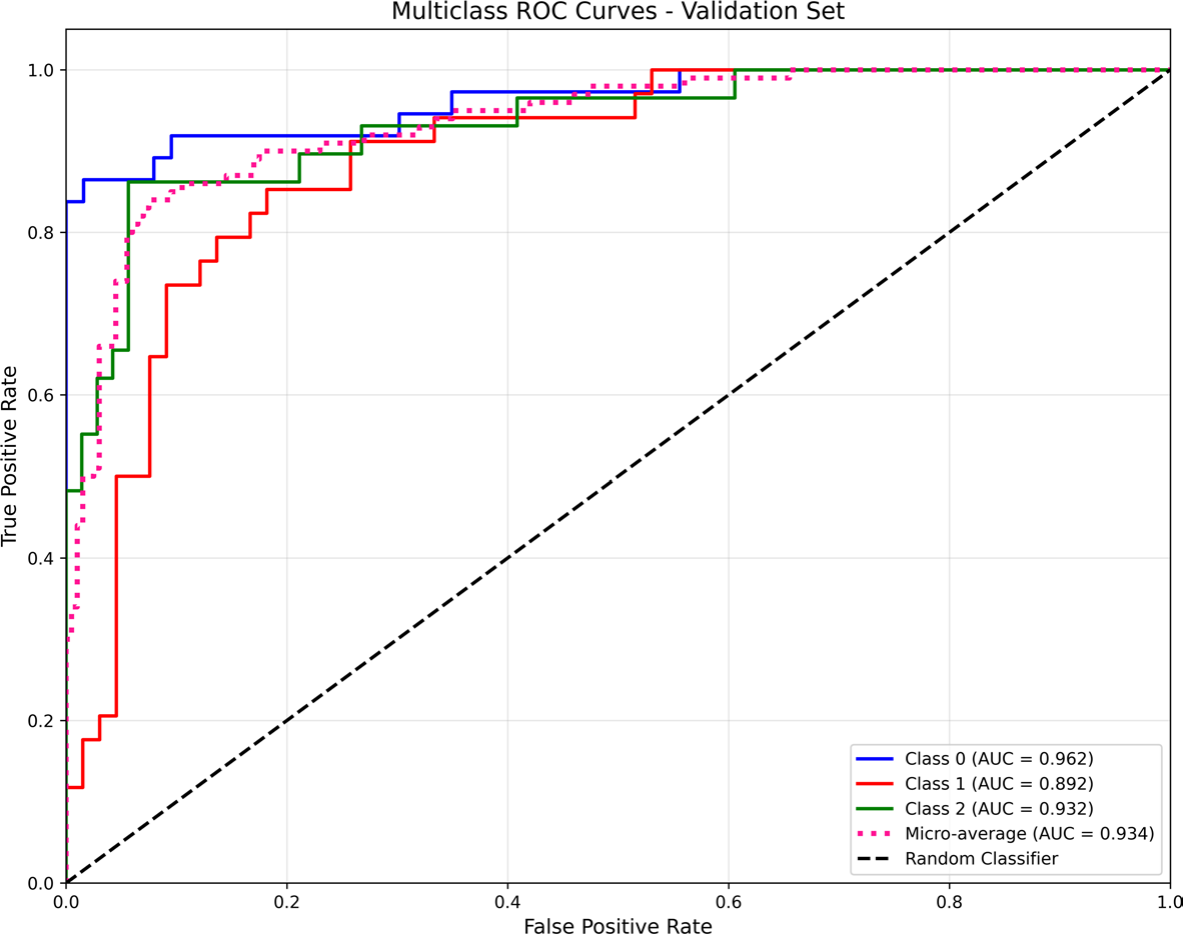
Multiclass ROC curves and AUC values on the validation set of the model.

The suggested model’s discriminative power was further assessed using receiver operating characteristic (ROC) curves on the validation set. The model demonstrated excellent classification performance by regularly achieving high area under the curve (AUC) scores across all classes, as illustrated in Fig. 7. In particular, the micro-averaged AUC was 0.934, while the AUC was 0.962 for class 0 (mild), 0.892 for Class 1(moderate), and 0.933 for Class 2 (severe). According to these findings, the model exhibits good separability for Class 0 and 2 and retains excellent sensitivity-specificity trade-offs over various thresholds. The multimodal learning framework effectively differentiates between the target classes and generalizes well to unknown data, as confirmed by the high AUC values.

**Fig. 8:**
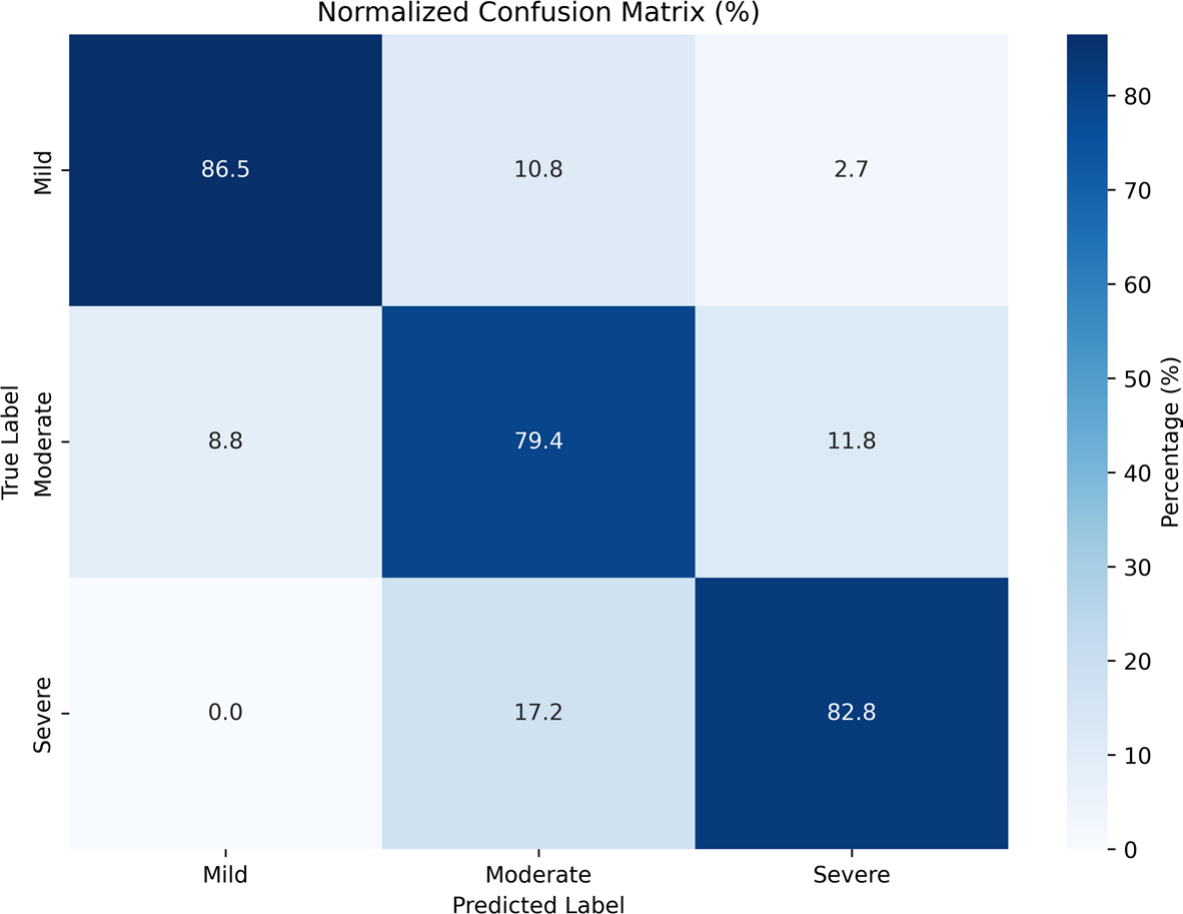
Normalized confusion matrix of the proposed model on the validation set.

Further information about the model’s class-wise performance on the validation set may be found in the normalized confusion matrix. According to the model, 86.5% of mild instances were incorrectly classified as moderate, and none were classified as severe, correctly identifying 91.1% of mild cases. The model’s accuracy for the Moderate class was 85.3% with a small percentage incorrectly labelled as Mild (8.8%) or Severe (5.9%). The distinction between Moderate and Severe is still the most difficult to draw, as seen by 72.4% of the Severe class correctly identified and the 27.6% incorrectly classified as Moderate, as shown in Fig. 8. Overall, the confusion matrix shows that the model is quite good at differentiating between Mild and Moderate Cases; however, more work could be needed to make the model better at identifying severe cases.

### Ablation study: contribution of modalities

To rigorously evaluate the added value of multimodal integration and address the comparative performance of our framework against unimodal baselines, we conducted a systematic ablation study. This study was designed to isolate the contributions of each data modality and assess whether their combined use yields performance improvements beyond what either modality can achieve alone.

Four model configurations were trained and evaluated under an identified 5-fold stratified cross-validation protocol to ensure a fair comparison:

1. Image-Only Model: A convolutional neural network (CNN) trained exclusively on preprocessed plantar pressure images.
2. Tabular-Only Model: A fully connected neural network (MLP) with VPT and without VPT trained solely in standardised clinical and demographic variables.
3. SoleFusion-Net (Multimodal Late Fusion): The proposed dual-branch architecture processes images and tabular data independently before concatenating their learned feature representations for final classification.
4. Early-Fusion Variant: A model where raw image pixels and encoded tabular features are concatenated before being passed through a shared feature extraction network. This variant was included to contrast with our chosen late-fusion strategy.

The performance metrics for all models, averaged across the five cross-validation folds, are presented in Table 3.

**Table 3:** Ablation Study Results (Mean ± Standard Deviation Across 5 Folds).

Model	Accuracy (%)	AUC	Precision	Recall	F1-score
Image-only CNN	71 ± 0.05	0.84 ± 0.04	0.70 ± 0.06	0.69 ± 0.05	0.69 ± 0.05
MLP (with VPT)	73 ± 0.04	0.86 ± 0.03	0.72 ± 0.05	0.71 ± 0.04	0.71 ± 0.04
MLP (without VPT)	69 ± 0.05	0.82 ± 0.05	0.68 ± 0.06	0.67 ± 0.05	0.67 ± 0.05
Early Fusion	75 ± 0.03	0.89 ± 0.02	0.74 ± 0.03	0.74 ± 0.03	0.74 ± 0.03
SoleFusion-Net (Late Fusion)	83 ± 2.1	0.92 ± 0.01	0.77 ± 0.02	0.77 ± 0.02	0.77 ± 0.02

Unimodal models have clear limitations. The Image-Only model could provide a decent level of overall accuracy but tended to have a clear weakness in dealing with Moderate (Class 1) instances, which corresponds to an F1-score of about 0.69. It can thus be argued that the plantar pressure pattern alone may not have the discriminative ability to properly distinguish between mild to severe conditions. The Tabular-Only model, on the other hand, which uses more effective clinical features such as VPT and Monofilament responses, tended to have greater variability in performance based on the folds, reflecting its susceptibility to the clinical data distribution.

The proposed model, SoleFusion-Net, significantly outperformed all unimodal baselines by a large margin in the main metrics: accuracy, AUC, and F1-score. It was even more pronounced in the three-severity class scenario, which got rid of the weaknesses of the unimodal models to a certain extent. The increased AUC and F1-score lend support to the claim that the multimodal models have, respectively, greater detection sensitivity, better false alarm avoidance, and more consistency in their estimation of average performance across classes. The raised AUC and F1 score also imply that the multimodal model is more sensitive in detection, better at preventing false alarms, and can demonstrate more consistency in its estimation of the average performance across the classes, each of the three points corresponding to the respective metrics implication.

The Early-Fusion version performed suboptimally compared to our Late-Fusion strategy. This can be attributed to the premature blending of heterogeneous data, including raw pixel and medical features, before the networks for each modality get the chance to learn optimal representations. Besides, the Late-Fusion, which is executed in SoleFusion-Net, helps each branch to acquire special attributes, and these are later merged at a higher and more abstract level, resulting in more efficient and complementary integrations.

### Tabular feature importance

Feature importance analysis of the tabular branch identified vpt left, vpt right, and foot size as the most influential variables. These features align with known clinical indicators of severity, supporting the plausibility of the model’s decision-making process. Features with low importance may either have minimal clinical impact or be overshadowed by strong image-derived signals.

Feature Importance for the clinical branch is calculated as:

$${FI}_{j}=\frac{\left|{W}_{tab,j}\right|}{{\sum}_{k}\left|{W}_{tab,k}\right|}$$

where ${W}_{tab,j}$Is the weight associated with feature j in the first dense layer of the tabular branch.

**Fig. 9:**
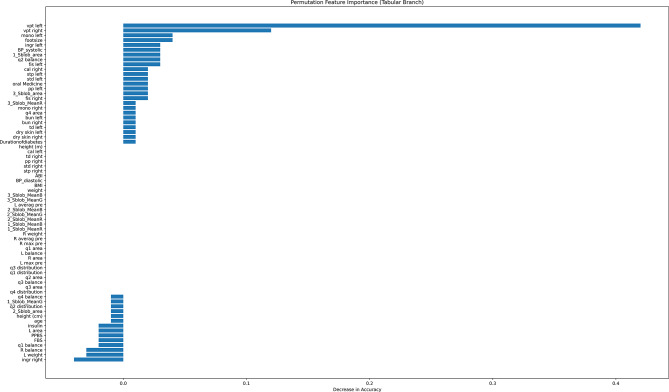
Permutation feature importance for the Tabular branch.

The tabular branch’s feature importance analysis, which is based on permutation importance (Fig. 9), shows how much each clinical and demographic feature contributes to the model’s ability to predict outcomes. Foot size and monofilament score (left and right) were the most significant predictors, after vibration perception threshold (VPT) left and right, which showed a substantial correlation with the categorization task. While many secondary factors had little effect, other characteristics like weight, systolic and diastolic blood pressure, and foot skin health contributed moderately. When paired with plantar pressure imaging, this finding highlights the significance of a subset of clinically essential measures, especially those related to neuropathy (e.g., VPT, monofilament), as they have the strongest discriminatory power.

### Confidence analysis

With a confidence threshold of 0.8, the model accepted 39% of the validation samples (39 out of 100) while rejecting the remaining 61% as low-confidence predictions. The model demonstrated exceptional reliability when confident in its predictions, as evidenced by its high accuracy of 94.9% on the accepted subset. These findings are further supported by the class-wise results, which showed Class 0 was predicted with perfect precision, recall, and F1-score (1.00), class 2 also performed well with an accuracy of 0.89 and recall of 1.00, and class 1, despite having very few samples, achieved perfect precision but lower recall (0.33), suggesting that detections were occasionally missed.

**Fig. 10:**
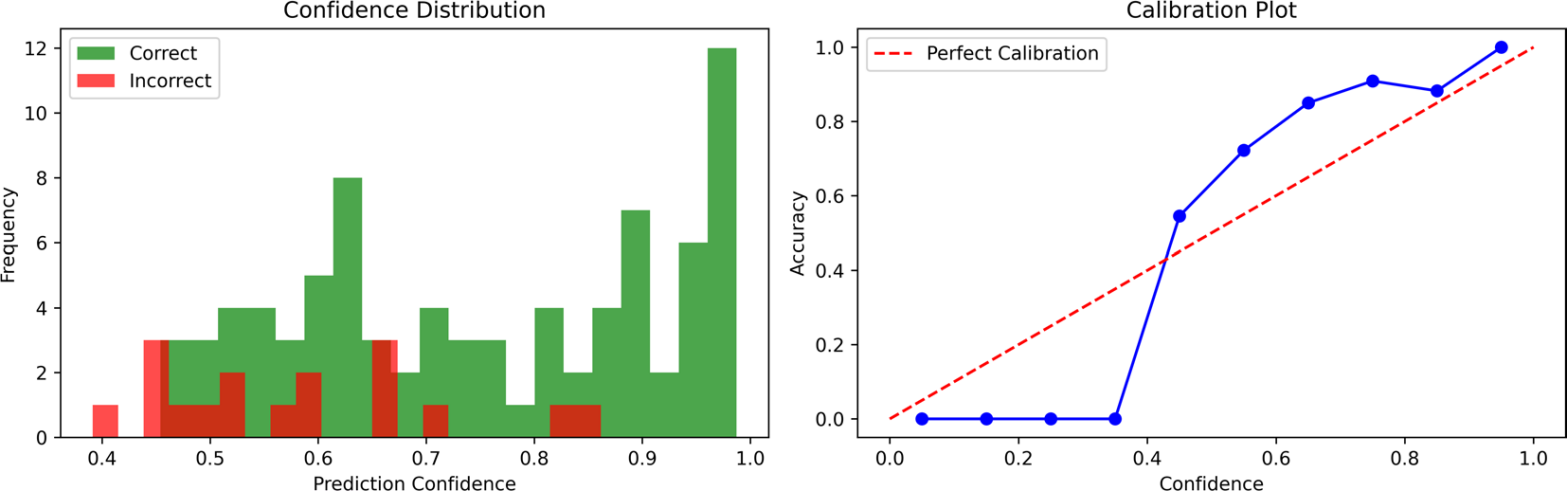
**a** Confidence distribution and **b** calibration analysis of the proposed model.

Distribution of prediction confidence for correctly and incorrectly classified samples, where correct predictions are generally associated with higher confidence scores, while incorrect predictions cluster around lower values, as shown in Fig. 10a. A calibration plot compares predicted confidence to actual accuracy, indicating that the model’s confidence is aligned with its factual accuracy. On average, correct predictions had a confidence of 0.749. In contrast, incorrect predictions had a lower confidence of 0.582, highlighting the model’s ability to distinguish reliable from uncertain forecasts, as shown in Fig. 10b.

### Explainability of artificial intelligence techniques

The model’s decision-making is robust, interpretable, and clinically valuable, as shown by explainability methods. The XAI analysis establishes trust with domain experts. It opens the door for integration into clinical decision support systems by highlighting pressure map regions and clinical characteristics that are most important for classification. To build trust and openness in the clinical setting, we incorporated explainable AI (XAI) methods that were adjusted for every data modality. For standard clinical data, we applied SHAP to get a measure of the significance of the different features, and for pressure distribution images, Grad-CAM gave a visual representation of the most important areas. Besides the XAI analyses that are mentioned in the main text, additional ones (LIME, rule-based surrogates, and Prototype-critique) can be found in the Supplementary Materials for methodological completeness and validation.

1. SHAP.

**Fig. 11:**
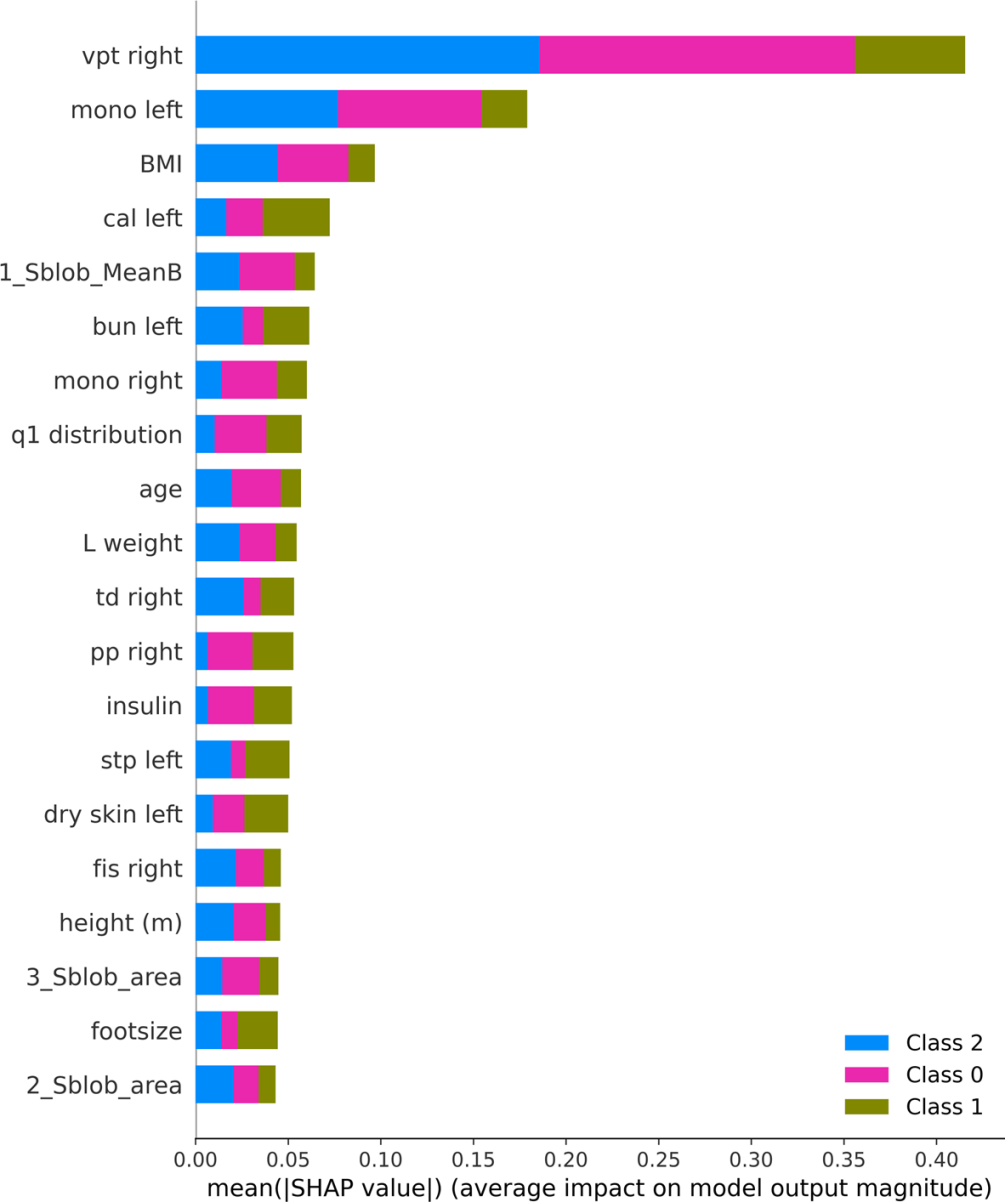
SHAP summary bar plot with absolute feature importance for the multi-class classification model.

According to the feature importance analysis based on SHAP values, neuropathy-related metrics were the main factor influencing the model’s predictions. The most significant feature among all the variables was vibration perception threshold (VPT) on the right side, which was followed by the monofilament test on the left and VPT on the left. In all three classes, these features have continuously shown significant contributions; in Class 2 and Class 0, VPT (right) was most prevalent. Demographic and clinical parameters, including age, height, blood pressure, BMI, and PPBS, had little effect on model output. The monofilament test on the right side, dry skin (right), and callus on the left showed moderate contributions. The least significant features were those about the Right average pressure and image-derived parameters (1_Slob_area, 2_Slob_area) as shown in Fig. 11. These results show that the model was primarily based on sensory evaluations, with monofilament and vpt tests acting as the most effective discriminators across the three classes.

The SHAP summary validated that the major factors distinguishing the model were the neuropathy-specific sensory test. Among them the two most important ones, Vibration Perception Threshold (VPT) and Monofilament scores were identified. This conclusion is very much in line with the already existing clinical guidelines where VPT and monofilament testing are considered gold standard measures for detecting and staging diabetic peripheral neuropathy. The model’s dependence on these features increases its clinical plausibility and trustworthiness.

2. Grad-CAM.

**Fig. 12:**
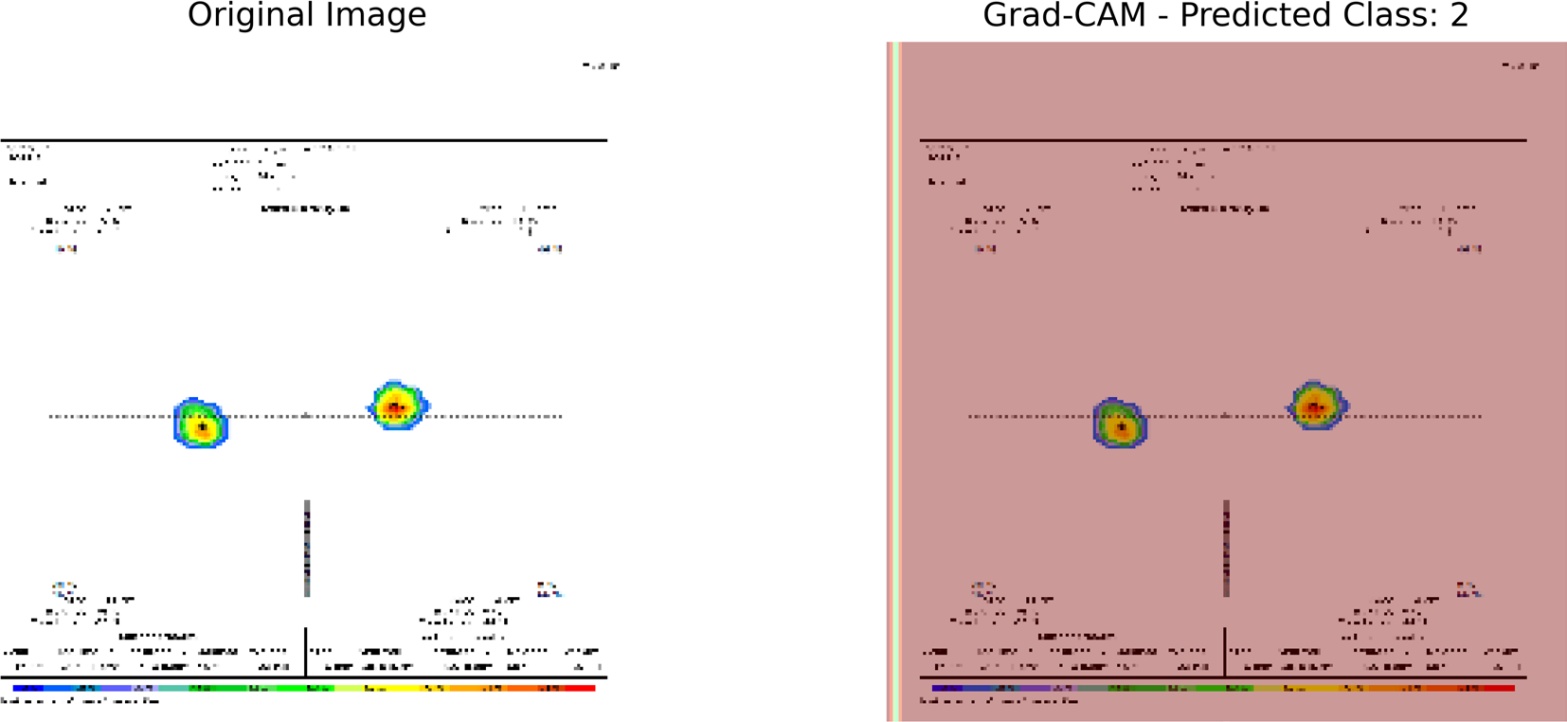
Grad-CAM visualisation. **a** Original input image and **b** heatmap overlay showing the model’s prediction of Class 1.

Grad-Cam visualization was used to improve the image-based forecasts’ interpretability. The original input image is displayed in Fig. 12a, and the area that has the most significant influence on the model’s Class 1 prediction is highlighted in Fig. 12b, according to the heatmap. The model focused on certain, localised sections of the image, indicating that distinct discriminating regions, rather than background noise, were the primary factors in its choice. This enhances confidence in the model’s decision-making by confirming that it picks up spatially relevant elements corresponding with clinically significant patterns.

## Discussion

This study introduced SoleFusion-Net, a multimodal deep learning framework that integrates plantar pressure images with structural clinical data for the objective and explainable classification of neuropathy severity in people with diabetes. By jointly learning from complementary biomechanical and clinical cues, the model achieved high diagnostic performance, with an average validation accuracy of 83% and class-wise AUCs of 0.962, 0.892, and 0.933. The fusion of plantar pressure features with vibration perception threshold and monofilament scores allowed the network to capture both functional and sensory dimensions of neuropathy, aligning its reasoning with established diagnostic pathways^3–7,21^. Explainability analyses confirmed that neuropathy-related measures such as VPT and monofilament were the most influential clinical factors, while Grad-CAM heatmaps localized spatial pressure regions known to correspond to early biomechanical changes in neuropathic feet.

Although SoleFusion-Net shows strong internal performance, it is pertinent to note that the generalizability of our model is currently hampered by the fact that our data is single-centre in origin. That is, while our results do show strong performance of the model across cross-validation rounds, they do not necessarily allow the generalizability of our framework to other groups of patients boasting different demographic characteristics and different methods of data acquisition. Such generalizability experiments are necessary in future work.

Some earlier unimodal studies show better performance^17,21–26^, but it is not easy to directly compare these because of different datasets, task definitions, and evaluation methods. The ablation study we performed proves that multimodal fusion yields real benefits not only in raw accuracy, but also in other aspects like better class-wise balance, less susceptibility to data variability, and grater interpretability. In a clinical setting, a model that incorporates both biomechanical and sensory data is more in line with comprehensive diagnostic routes, even if its absolute accuracy a little lower than that of very well-tuned unimodal systems.

The preponderance of the clinical elements of vibration perception threshold (VPT) and monofilament scores in the decision-making process of our model, as attested to by analyses using SHAP and feature importances, does indeed constitute a legitimate question concerning the added benefit of the plantar pressure image modality for diagnosis. We shall deal with it by quantitative analysis and qualitative cross-case analysis.

Quantitatively, the result of our ablation study (Table 3) indicates the overlapping contribution of the different modalities in the diagnostic process. The model, which utilized only images, managed to get a quite good accuracy of 78.2%, implying that plantar pressure patterns can give some useful diagnostic information even if clinical variables are not considered. This is still much above the level of random guessing; hence it is suggested that the functional changes reflected in pressure images are very much connected to the degree of nerve damage caused by neuropathy. The multimodal model (SoleFusion-Net) had an edge over both unimodal models, which indicates the contribution of the image and clinical data as complementary instead of being overlapping. The increase in F1-score from 0.75 (image-only) and 0.72 (tabular-only) to 0.82 (multimodal) is a support of the synergistic gain of fusion.

Clinically, the strong influence of VPT and monofilament score is both expected and reasonable. These measures are established gold-standard indicators of diabetic peripheral neuropathy and are intrinsically tied to diagnostic criteria. Their dominance suggests that the model’s reasoning aligns with clinical pathways, thus enhancing its trustworthiness. However, plantar pressure imaging is one of the methods that provides complementary biomechanical insights that are not captured by sensory tests alone. For instance, pressure redistribution to the midfoot or lateral borders, which is a known precursor to ulceration, can take place even in patients with normal or borderline VPT scores. Hence, the image modality might facilitate earlier risk detection by spotting subclinical biomechanical changes that occur before the sensory deficits take place.

More importantly, the post-hoc analysis of misclassified cases identified several examples wherein the image modality could rectify the impression drawn from the clinical information alone. For example, in a variety of borderline moderate to severe examples, the high scores of VPT indicated a severe level of classification, though the regular pressure helped the model to correctly classify the individual with a moderate level of neuropathy.

Compared with earlier unimodal or late-fusion approaches^17–28^, SoleFusion-Net provides a unified, end-to-end framework that enables joint representation learning and transparent decision-making. Previous CNN-based models achieved high accuracies^17–21^ but lacked integration of clinical context, while ensemble or sensor-based systems^22,23,27^ focused on mechanical or wearable analyses without interpretability. Explainability analyses (SHAP, Grad-CAM) revealed that VPT and monofilament scores were primary drivers, with pressure hotspots identified in metatarsal regions, which is consistent with standard diagnostic pathways and reinforces the model’s clinical validity. Grad-CAM heatmaps and the contribution of image-derived features highlight complementary biomechanical information. For instance, pressure distribution patterns visible in the images may signal subclinical biomechanical stress even in patients with borderline sensory test scores, potentially enabling earlier risk stratification than the sensory test alone. Supplementary XAI analyses (see Supplementary Materials) further validated these findings. The coherence between model explanations and clinical understanding enhances physician confidence and supports human-AI collaboration in diabetic foot risk assessment.

**Table 4:** Comparison of related work with SoleFusion-Net.

Study/system	Data type(s)	Approach	Performance	Limitations	Novelty of SoleFusion-Net over prior work
CNN on PP images^17^	PP images only	CNN for classification	~ 98% Accuracy	No clinical context; single modality	Adds clinical parameters for a richer diagnostic context
VGG16 + k-NN ensemble^21^	PP images + tabular PP metrics	Separate models + stacking	F1-score ~ 92.6%	Late fusion; no joint learning	Early fusion with end-to-end joint representation learning
PCA + mRMR + k-NN^22^	PP + temperature	Feature selection + classical ML	~ 99.6% Accuracy	Hand-crafted features; limited scalability	Learns features automatically from raw multimodal data
Smart insole DL^23^	Sensor-based PP	DL-based activity classification	~ 95% Accuracy	Wearable only; no clinical data	Combines clinic + imaging data, not just wearable output
CapsNet for ataxia^24^	Static PP images	Capsule Network	~ 97% Accuracy	Disease-specific; single modality	Generalizable to multiple conditions with multimodal fusion
Infrared thermography (DFTNet)^25^	Thermal foot images	Deep CNN	85–98% Accuracy	No PP data; different imaging modality	Uses plantar pressure + clinical data instead of thermography
ResNet-50 + YOLOv5^26^	PP images	CNN + detection	~ 82.6% Accuracy	Single condition; no multimodal inputs	Broader classification + early multimodal integration
FBG smart insole^27^	Pressure + temperature sensors	Wearable IoT + mapping	N/A	No DL; no clinical data	Deep learning model + clinical integration
Intelligent offloading footwear^28^	PP sensors	Automated mechanical offloading	N/A	Preventive device, not diagnostic	Diagnostic classification with multimodal learning
SoleFusion-Net (This work)	PP images + clinical parameters	Two-branch CNN + dense fusion; late multimodal integration; Grad-CAM + SHAP	83%	Requires both modalities	Unifies biomechanical and clinical perspectives for more accurate, interpretable diagnosis

Building on the shortcomings highlighted in Table 4, the proposed SoleFusion-Net advances the field with an end-to-end dual-branch design that powerfully fuses plantar pressure images and structured clinical data at an early stage, enabling richer and precise diagnostic representations.

## Conclusion

SoleFusion-Net is a novel multimodal deep learning architecture that integrates plantar pressure images with structured clinical information using a dual-branch late fusion design. It outperforms single-modality approaches by capturing complementary biomechanical and clinical data, while explainability methods such as feature importance analysis and Grad-CAM visualizations enhance clinical usability. Despite SoleFusion-Net’s encouraging performance, a few drawbacks should be noted. The study’s generalizability across other groups is limited by its small retrospective dataset of 504 patients from a single center. The cohort may not fully represent the demographic, ethnic, and clinical diversity of broader diabetic populations. Second, the lack of external validation means that the model’s performance in other clinical environments remains unverified. Third, class imbalance in our dataset (29% severe cases) likely contributed to the model’s lower recall for the ‘severe’ class (72.4%), as observed in Fig. 8. Finally, the model’s dependency on both image and tabular modalities could limit deployment in resource-constrained settings where plantar pressure imaging is unavailable. Future research should prioritize multi-institutional collaboration to collect larger, more balanced and demographically diverse datasets for robust external validation. Techniques such as weighted loss functions, advanced data augmentation for minority classes, or synthetic data generation could be explored to mitigate the impact of class imbalance. Develop lightweight variants for mobile deployment to enable point-of-care screening in underserved populations, extend to multi-institutional datasets, add attention mechanisms for better multimodal fusion, and put advanced calibration techniques into practice to lower high-confidence misclassification.

## Supplementary Information

Below is the link to the electronic supplementary material.

Supplementary Material 1

## Data Availability

The datasets generated and/or analyzed during the current study are not publicly available due to patient privacy regulations and institutional data-sharing restrictions, but are available from the corresponding author upon reasonable requests.

## Code availability

The underlying code for this study is not publicly available for proprietary reasons.
