# Supplementary material for: SoleFusion-Net: an explainable multimodal deep learning framework for diabetic foot syndrome classification in type II diabetes mellitus
Source: Sci Rep. 2026 Apr 3;16:15973. doi: 10.1038/s41598-026-42207-6 (PMC13195104; doi:10.1038/s41598-026-42207-6)
Supplement: Supplementary file 1 — Supplementary Material 1 [file 41598_2026_42207_MOESM1_ESM.pdf]

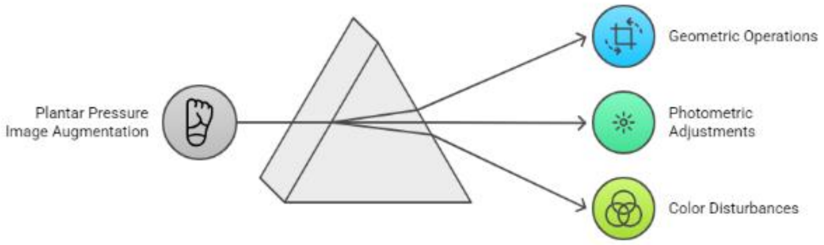

Supplementary Figure S1: Process of Augmentation

The preprocessing pipeline included geometric augmentations (random cropping, flipping, small rotations), photometric adjustments (brightness, contrast, gaussian noise), and color transformations (Grayscale, histogram equalization, HSV shifting). These were implemented as custom TensorFlow layers for GPU-accelerated, reproducible augmentation.

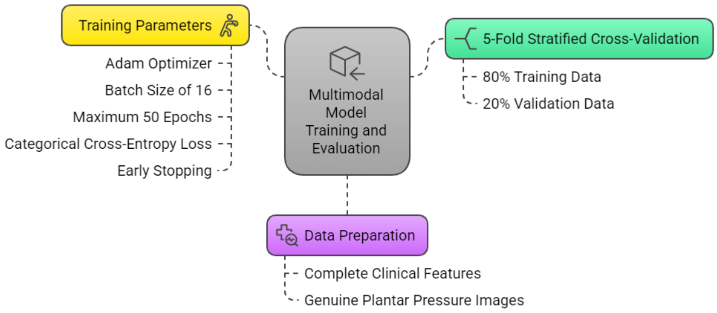

Supplementary Figure S2. Flowchart for Training Strategy

The figure illustrates the training and evaluation of the model were carried out through 5-fold stratified cross-validation (for each fold, 80% was used for training and 20% for validation), thus keeping the distribution of classes intact. Adam optimizer with a batch size of 16, categorical cross-entropy loss, and early stopping over a maximum of 50 epochs were used in training. In order to ensure data consistency, only complete multimodal records were included.

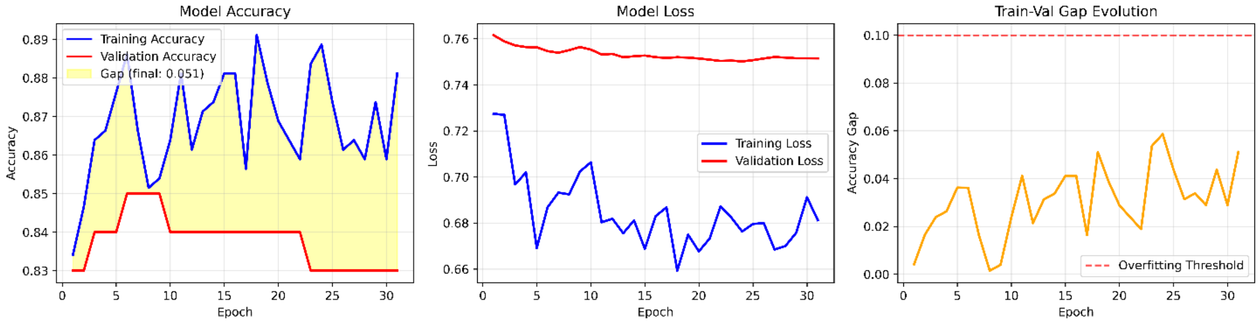

Supplementary Figure S3. The graph illustrates Model accuracy across epochs, Model Loss across Epochs, and Learning rate adjustment during training.

The model showed strong generalization with only a ~4.4% train-validation gap, minimized via cross-validation and regularization. This confirms robustness without significant overfitting.

Explainability of Artificial Intelligence Techniques

1. LIME

Analyses of local interpretability were conducted to comprehend how the model made a single prediction. The feature-level contributions to the classification of a representative sample are shown in Figures 15 (a) and (b). Class 0 was predicted by the model with a high probability (0.84), while Class 1 and Class 2 had 0.15 and 0.01, respectively, while std right, callus left, bunion left, and vpt right. Td right contributed adversely, decreasing the chance of Class 1. Variables like q1 area, bun right, height (m), PPBS, and q4 balance contributed positively towards Class 1, as seen in Supplementary Fig S3(a). The bar representation in Supplementary Fig S3(b) further validates this, identifying q1 area as the most significant positive contributor, whereas the std right and callus left were dominating negative contributors. These findings collectively show how local explanation techniques can separate feature effects on individual predictions, making the model's classification results easier to understand.

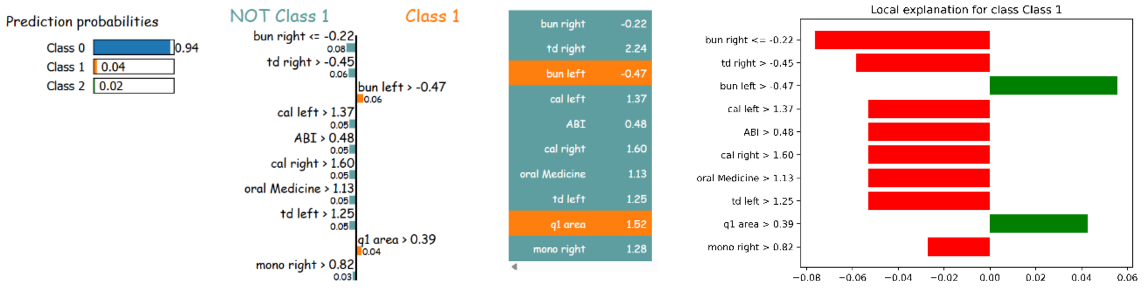

(a) (b)

Supplementary Figure S4. LIME decision plot for (a) the decomposition of Class Probability and (b) the Representation of feature contribution

2. Rule-based Surrogates

The decision tree highlights the hierarchical feature splits that determine classification into Classes 0,1, and 2. According to the root node, the most significant discriminator is vpt right: samples with values ≤ 0.428 are steered to the left, whereas samples with higher values are steered to the right (primarily Class 2). Additional splits on the left side are on the q1 area, dry skin on the right, and the R average before Class 0 and Class 1 are separated. While supplementary variables like q1 area, R area, and duration of diabetes aid in differentiating between borderline patients, the tree structure reveals that vpt right is the main decision driver, as shown in Supplementary Figure S5. With further support from area-based and clinical features, this interpretable model highlights the predominance of metrics associated with neuropathy.

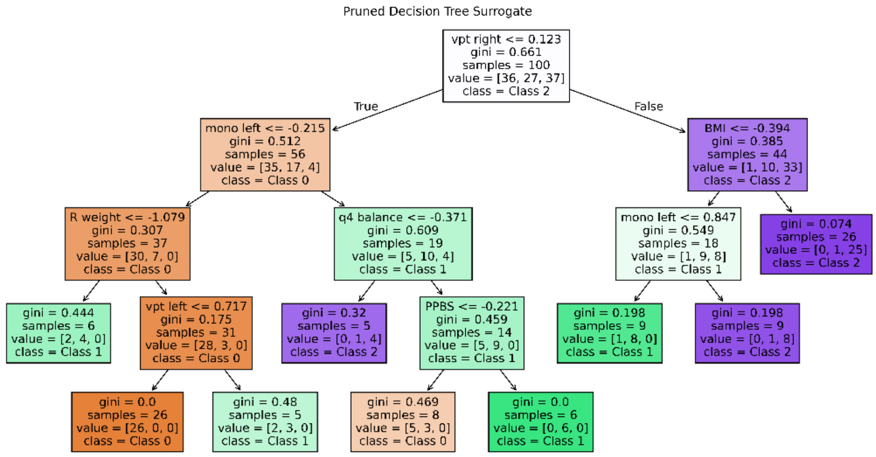

Supplementary Figure S5. Decision tree visualization showing hierarchical feature splits for classification.

3. Prototype and Criticism-based Explanations

In the embedding space, prototype and critique analysis were further investigated. Prototypes (green) cluster close to the dense core region, reflecting typical examples, while critiques (red) lie at the periphery, suggesting atypical or boundary cases that test the model. Supplementary Figure S6(b) displays a PCA projection of all validation samples. This distinction demonstrates the mode's ability to recognise outliers and representative patterns. In addition, the validation set pairwise distance matrix is shown in Supplementary Figure S6(b), where brighter parts show different samples and darker blocks show more similar samples. When taken as a whole, these analyses show that prototypes capture key trends in the data, whereas critiques highlight edge cases and diversity, enhancing the models' interpretability and robustness evaluation.

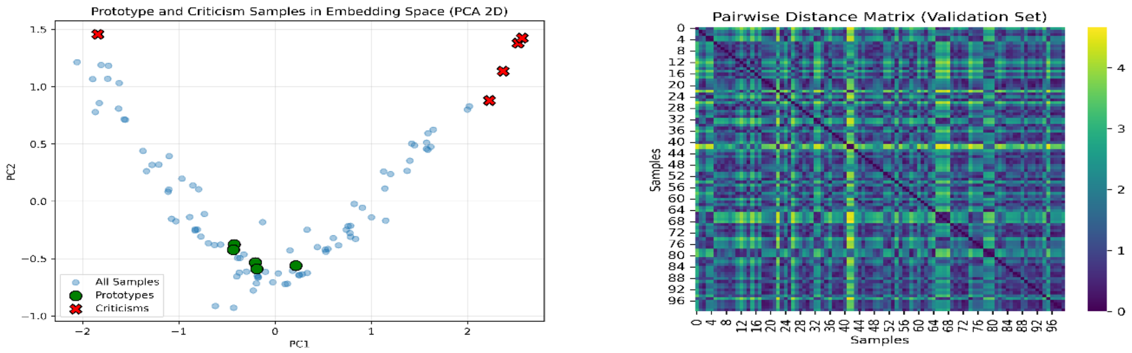

(a) (b)

Supplementary Figure S6: Prototype and criticism analysis using (a) PCA-based 2D embedding and (b) Pairwise distance matrix
